# Association of vitamin A supplementation with immune-related allergic diseases: A meta-analysis

**DOI:** 10.3389/fnut.2022.984161

**Published:** 2022-11-18

**Authors:** Jingqiu Su, Tian Li, Hua Pan

**Affiliations:** ^1^Department of Dermatology, The Fourth Affiliated Hospital of China Medical University, Shenyang, China; ^2^School of Basic Medicine, Fourth Military Medical University, Xi’an, China

**Keywords:** vitamin A, allergic diseases, wheeze, meta-analysis, systematic review

## Abstract

**Background:**

Allergic diseases are type I hypersensitivity reactions mediated by various allergens. The most common allergic diseases include allergic rhinitis, allergic asthma, allergic dermatitis, and allergic conjunctivitis. The incidence of allergic diseases has been increasing in the recent past, and allergen avoidance and adoption of desensitization treatment can significantly decrease the incidence of allergic diseases. Previous studies have explored the association between vitamin A supplementation and allergic diseases; however, the results are inconsistency. The aim of the present study was to evaluate the association between vitamin A supplementation and allergic diseases, with a focus on atopy and wheezing.

**Methods:**

Articles reporting randomized controlled trials (RCTs) on the association of vitamin A supplementation and allergic diseases were retrieved from PubMed, Embase, Web of science, and China National Knowledge Infrastructure database from inception of to November 15, 2021. STATA 12.0 software was used for meta-analysis, sensitivity analysis and analysis of publication bias.

**Results:**

Seven studies comprising 2201 participants met the inclusion criteria and were included in the meta-analysis. The findings showed that vitamin A supplementation was associated with increased risk of atopy in young females compared with the placebo [RR = 1.70, 95% confidence interval (1.20, 2.41), *P* = 0.171, *I*^2^ = 43.4% fixed effect model]. The frequency of delayed atopy among adults was associated with vitamin A supplementation (MD = 0.46, 95% CI = 0.04, 0.88). Analysis showed no significant association between vitamin A supplementation with incidence of wheezing in children [RR = 1.40, 95% CI (0.49, 3.98), *P* = 0.018, *I*^2^ = 82.1% random effect model]. Sensitivity and publication bias analysis showed that each individual study did not affect the combined results and there was no significant publication bias among the studies.

**Conclusion:**

The findings showed that vitamin A supplementation is associated with increased risk of atopy but no correlation was observed with the incidence of wheezing. The results of this meta-analysis provide evidence for effective management of fibrosis. More studies should be conducted to verify the results.

## Introduction

Allergic diseases are type I hypersensitivity reactions mediated by various allergens. Allergic rhinitis, allergic asthma, allergic dermatitis, and allergic conjunctivitis are the most common allergic diseases. Allergens can be divided into two categories: perennial allergens, and seasonal allergens. Perennial allergens mainly include dust mites, mold, cockroaches, and other indoor allergens whereas seasonal allergens include pollen and other outdoor allergens. The incidence of allergic diseases has been increasing in recent years and these diseases markedly affects the quality of life of patients.

Allergen detection is an important step in diagnosis of allergic diseases. The allergen avoidance method and desensitization treatment are effective in treatment of allergic diseases. Conventional methods used for treatment of allergic diseases include use of allergy medications and hormones, which have adverse effects. Bronchial asthma (asthma for short) is a common chronic disease that affects children aged 5–14 years worldwide (1). Bronchial asthma is an allergic disease that attacks various cells and cell components. The incidence of asthma, eczema, allergic rhinitis and other allergic diseases has been increasing yearly, especially in children (2, 3). The incidence of allergic diseases is highly correlated with immune imbalance, inflammatory reaction caused by allergen stimulation and family genetic factors (4). The association between allergic diseases and fibrosis has been studied for several years; however, the results of different studies are not consistent.

Vitamin A deficiency (VAD) is a worldwide nutritional and public health concern (5). Studies report that the increased incidence of allergic diseases is associated with changes in serum vitamin A levels induced by dietary changes (6, 7). Previous findings indicate that the levels of serum vitamin A and dietary intake in patients with asthma were significantly lower than those in healthy people. In addition, levels of serum vitamin A in patients with severe asthma were significantly lower than those in patients with mild asthma. Low levels of vitamin A are correlated with severe asthma attacks (8, 9). Some studies report that vitamin A can regulate immunity, induce immune function, has antioxidant activity, maintain the integrity of the airway epithelium, promote normal development of airway smooth muscle, and reduce airway hyperresponsiveness. Vitamin A supplementation may play a certain protective role in allergic diseases (10, 11). These findings indicate that vitamin A is associated with the incidence of allergic diseases.

Studies have been conducted to explore the effect of vitamin A on allergic diseases, but the results are not conclusive. Therefore, the presented study was conducted to evaluate the association between vitamin A and incidence of allergic diseases.

## Materials and methods

### Search strategy

Randomized controlled trials published in PubMed, Web of Science, Embase, and China National Knowledge Infrastructure databases from inception of the database to Nov 15, 2021 were retrieved using the following search terms: (1) Vitamin A; (2) allergy; (3) wheeze; (4) atopy; (5) allergic diseases; (6) allergic; (7) immune; (8) supplement; (9) supplementation. The search strategy comprised medical subject headings (mesh) and text words joined by the Boolean operator “and.” This meta-analysis was registered in INPLASY platform (202240032). The methodology is referred to previous publications (12–16).

A comprehensive search was conducted in the databases without restrictions on language or publication status. The search was restricted to studies comprising human participants. Articles from the bibliographies of retrieved articles were included to find other relevant studies not found through the search strategy.

### Study selection

A comprehensive review of the retrieved articles was conducted to ensure that they met the following inclusion criteria: (1) study design was double-blind, randomized, placebo-controlled trials; (2) the intervention was vitamin A or placebo; (3) the outcome was allergic diseases, including atopy, wheezing, etc.; (4) the full text was available for reference.

The exclusion criteria in this study were: (1) studies on other non-human subjects; (2) studies that compared other interventions; (3) studies that had insufficient data; (4) comments, abstracts, reproduction of publications.

### Data collection and quality assessment

Two reviewers (JQS and TL) independently reviewed detailed information including the titles, abstracts, and full-text articles of the retrieved studies. Any differences between the two reviewers were resolved through discussion with a third reviewer (HP). The following data parameters were extracted from the studies: name of main author, publication year, country, participants, number of participants in each group, characteristics of intervention, and outcome. The JADAD scale was used to evaluate the methodological quality of the selected randomized controlled trials. The JADAD scale comprised three items with a score of 0–5 points as follows: (1) generation method of the random grouping sequence (0–2 points); (2) Double-blind method: the specific protocol of double-blind implementation was described and considered appropriate (0–2 points); (3) Withdrawal and loss of subjects to follow-up: the number of cases that withdrew from the study and loss to follow-up and reasons for withdrawal were described in detail (0–1 point). Egger’s test and funnel plot was generated assess the publication bias in included studies.

### Statistical analysis

Heterogeneity of included studies was determined by chi-squared test and *I*^2^ statistics: values of 0–25% represented low heterogeneity, 26–75% moderate heterogeneity, and values greater than 75% indicated significant heterogeneity. Data were considered highly heterogeneous if the chi-squared test presented a *P*-value < 0.10 and *I*^2^ > 50%. The random effect model was used for analysis of studies if *P* < 0.05 or *I*^2^ > 50%, otherwise the fixed effect model was used to conduct the analysis. The funnel plot was visually analyzed to assess publication bias. Sensitivity analysis was conducted to explore the potential sources of heterogeneity. Sensitivity analysis was performed by deleting one study at a time to evaluate the impact of individual results on the overall analysis. All statistical analyses were conducted using STATA software version 12.0. All statistical analyses were two tailed, and *P* < 0.05 indicated statistical significance.

## Results

### Search process

**FIGURE 1:**
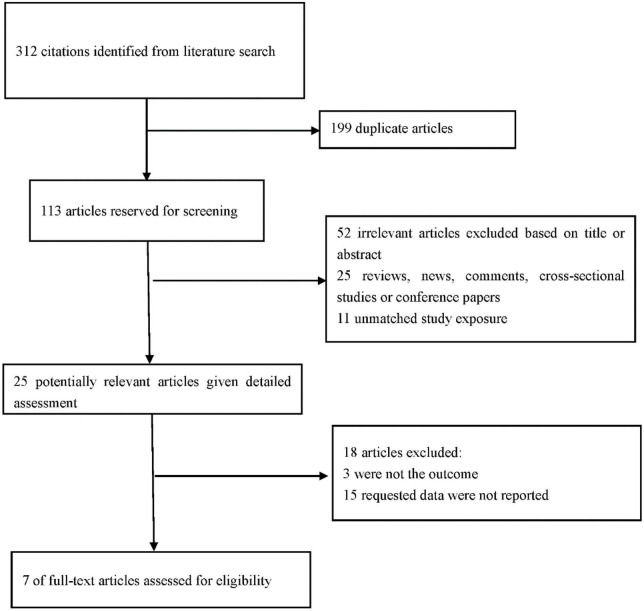
Flow chart showing the process for selection of eligible studies.

A total of 312 articles were retrieved from PubMed, EMBASE, web of science and CNKI databases through the initial search. Subsequently, 113 articles were retained after the first screening. An additional 88 records were excluded after reviewing the titles and abstracts of retrieved articles since they were review articles, case reports, letters, comments, or editorials, resulting in 25 eligible articles. A total of 18 articles were further excluded for various reasons, including different research designs or insufficient data. Finally, 7 studies comprising 2,201 participants met the inclusion criteria and were included in this meta-analysis (17–23). PRISMA guidelines were followed in conducted this meta-analysis the reasons for excluding studies were presented (Figure 1).

### Characteristics of included studies

The main characteristics of the eight studies included in this review are presented in Table 1. These studies comprised 2,201 participants [1,044 patients (experimental group) and 1,157 patients (control group)]. The sample size in the included studies ranged from 24 to 1,478.

**TABLE 1:** Characteristics of eligible studies.

References	Country	Outcome	Number of participants (Trial/control)	Age of the subjects	Intervention type	JADAD score
Fuller et al. (20)	US	Frequency of delayed atopy	12/12	Men aged 19–39	β carotene, 30 mg/d	7
Bogden et al. (21)	US	Frequency of delayed atopy	29/26	Individuals aged 59–85	β carotene, 0.75 mg/d	6
Herraiz et al. (22)	US	Frequency of delayed atopy	15/16	Men aged 55–79	β carotene, 30 mg/d	7
Wolvers et al. (23)	Netherlands	Frequency of delayed atopy	31/31	Individuals aged 40–80	β carotene, 12 mg/d	6
Kiraly et al. (17)	Guinea	Rapid atopy, rapid atopy in young female participants	131/132	Healthy babies for 6 and 9 months	Vitamin A, 1,00,000 U	5
Kiraly et al. (18)	Guinea	Rapid atopy, rapid atopy in young female participants, wheezing	76/76 or 78/210	Low birth weight infant	Vitamin A, 25,000 U	6
Aage et al. (19)	Guinea	Rapid atopy, rapid atopy in young female participants, wheezing	724/706 or 748/730	Normal birth weight infant	Vitamin A, 50,000 U	6

### Results of the meta-analysis

#### Association between vitamin A supplementation and atopy

**FIGURE 2:**
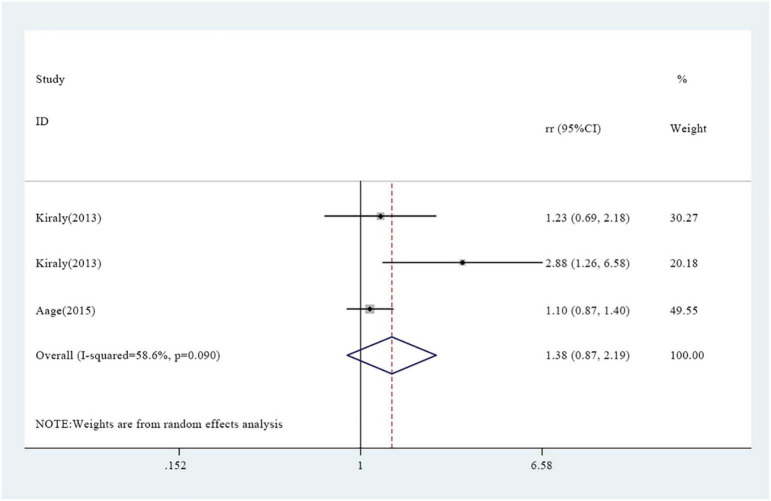
Association between vitamin A supplementation and rapid atopy.

**FIGURE 3:**
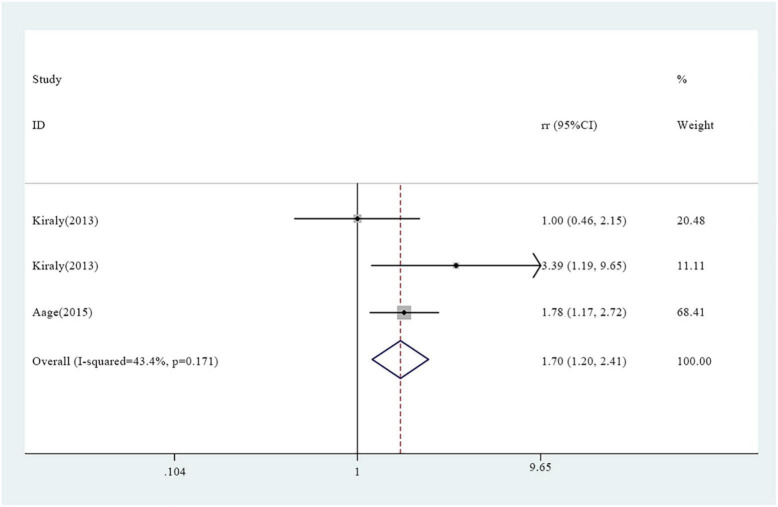
Association between vitamin A supplementation and rapid atopy among young female participants.

A total of seven studies reported association between vitamin A supplementation and atopy, including rapid atopy and delayed atopy. Notably, three studies reported findings on rapid atopy whereas four studies reported the frequency of delayed atopy. The overall results on rapid atopy showed that the incidence of rapid atopy in the experimental group was not significantly different from the incidence of the control group [RR = 1.38, 95% confidence interval (0.87, 2.19), *P* = 0.090, *I*^2^ = 58.6% random effect model) (Figure 2). Further analysis was conducted based on gender and the results revealed that the incidence of rapid atopy in young females in the experimental group was significantly higher relative to that of the control group [RR = 1.70, 95% confidence interval (1.20, 2.41), *P* = 0.171, *I*^2^ = 43.4% fixed effect model] (Figure 3). The results on delayed atopy showed that the frequency of delayed atopy in the experimental group was markedly higher than that of the control group (MD = 0.46, 95% CI = 0.04, 0.88).

### Association between vitamin A supplementation and wheezing

**FIGURE 4:**
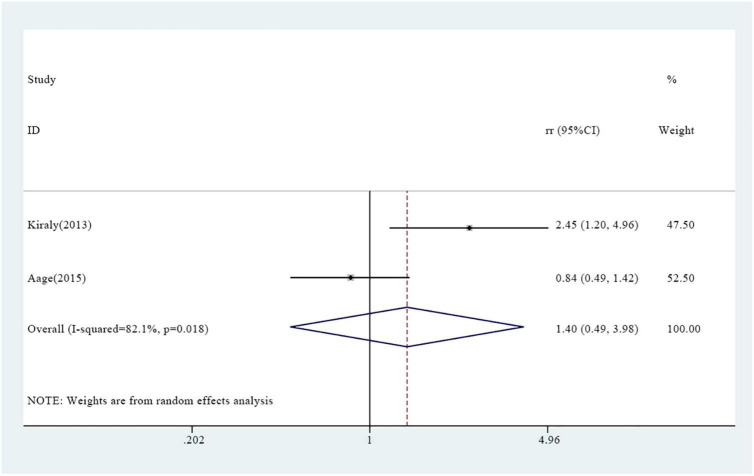
Association between vitamin A supplementation and wheezing.

Two studies revealed an association between vitamin A supplementation and wheezing. The overall results showed that the incidence of wheezing in the experimental group was not significantly different from that of the control group [RR = 1.40, 95% CI (0.49, 3.98), *P* = 0.018, *I*^2^ = 82.1% random effect model] (Figure 4).

### Quality of included studies

Jadad assessment tool was used to evaluate the methodological quality of included studies. All 7 articles were evaluated from three aspects: “random grouping sequence generation method,” “allocation hiding” and “blinding method.” The results showed that the 7 articles were of high quality (Table 1).

### Sensitivity analysis and publication bias results

Sensitivity analysis showed that exclusion of one study at a time did not alter significantly the combined risk ratio (RR) (Supplementary Figures 1–3). Visual inspection of funnel plots showed that there was no significant asymmetry (Supplementary Figures 4–6). No evidence of publication bias was observed through Begg’s and Egger’s test.

## Discussion

In this study, the findings showed that the incidence of rapid atopy among young female participants in the vitamin A supplementation group was higher than that of the control group. In addition, the frequency of delayed atopy in the experimental group was higher than that of the control group. However, the incidence of wheezing in the experimental group was not markedly different from that of the control group. The results of our study have important clinical significance since they indicated that reasonable supplementation of vitamin A is important for both children and adults.

The findings did not show any association between vitamin A supplementation and wheezing. However, some previous studies reported an association between vitamin A and wheezing. For instance, previous findings indicated that a low dose of vitamin A supplementation in early life is a protective factor for skin and respiratory allergic diseases (24, 25). Moreover, Parr et al. reported that administration of high doses of vitamin A during pregnancy and in children and adults with significantly increased the risk of asthma (26). These findings indicate varying effects of vitamin A supplementation dose and intervention stage on allergic diseases. Some studies report that intake of diet with vitamin A supplementation is an important protective factor of allergic diseases. The vitamin A plays a significant role in promoting normal lung development, improving lung function and alleviating oxidative damage in early life (27, 28). Therefore, studies should further explore the effects of vitamin A supplementation on wheezing in children.

The mechanism of the risk of atopy caused by higher doses of vitamin A supplementation has not been fully elucidated. Schuster et al. reported that large doses of VAS can inhibit Th1 function, promote Th2 cytokine expression, and upregulate expression of IL4, IL5, IgE and eosinophils, but aggravate immune disorders (29). This finding shows that the immune response varies with gender, age and the dose administered. The results of these clinical studies are consistent with the findings reported in most animal studies (30, 31), indicating that reasonable supplementation of vitamin A supplementation in early life plays an important role in inducing the immune system, immune regulation and reduction of disease incidence and mortality in offspring.

The present meta-analysis had some limitations. First, baseline data were inconsistent across some articles. Second, retrieval of studies published in only one language, different dosages, varying dosage forms and different study regions where vitamin A supplementation was conducted may lead to biased results. Third, only seven studies were included in this meta-analysis and the results should be further verified by including more studies conducted using different populations.

In summary, the findings of this meta-analysis indicate that vitamin A supplementation may increase the incidence of rapid atopy and increase the frequency of delayed atopy. The results provide practical and valuable insights for vitamin A supplementation. Additional prospective large-scale studies conducted in more countries/populations should be analyzed to elucidate the underlying mechanisms underlying the effect of vitamin A supplementation and atopy.

## Data availability statement

The original contributions presented in this study are included in the article/Supplementary material, further inquiries can be directed to the corresponding author/s.

## Author contributions

JS drafted the manuscript, generated the figures for manuscript, and conducted literature review. TL performed literature review and revised the final manuscript. HP provided supervision and contributed to the conceptualization of the review. All authors read and approved the final version of the manuscript for publication.
