# Supplementary figures and images for: Association of vitamin A supplementation with immune-related allergic diseases: A meta-analysis
Source: Front Nutr. 2022 Nov 18;9:984161. doi: 10.3389/fnut.2022.984161 (PMC9715979; doi:10.3389/fnut.2022.984161)

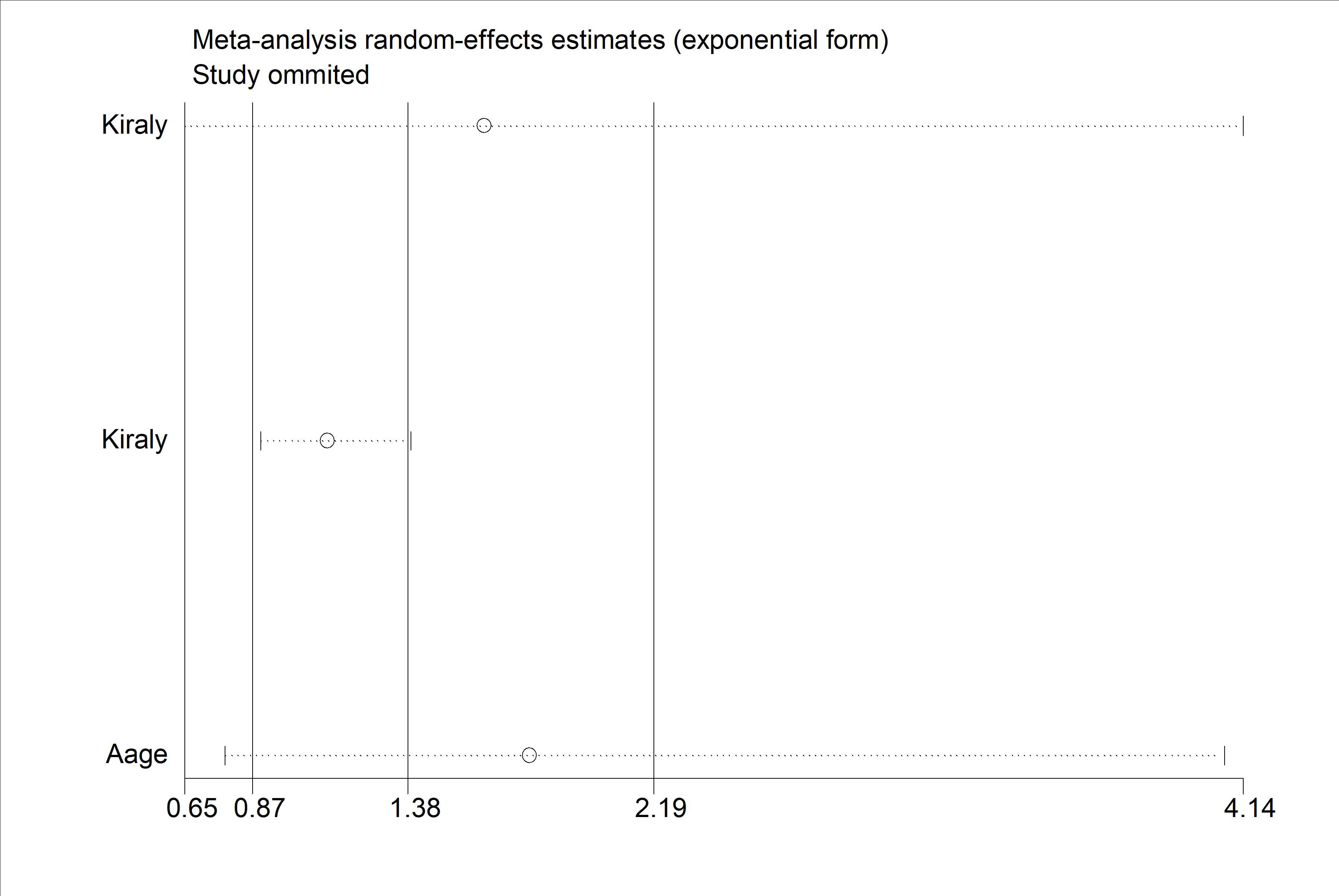

Supplement: Supplementary file 2 [file Image_1.jpg]

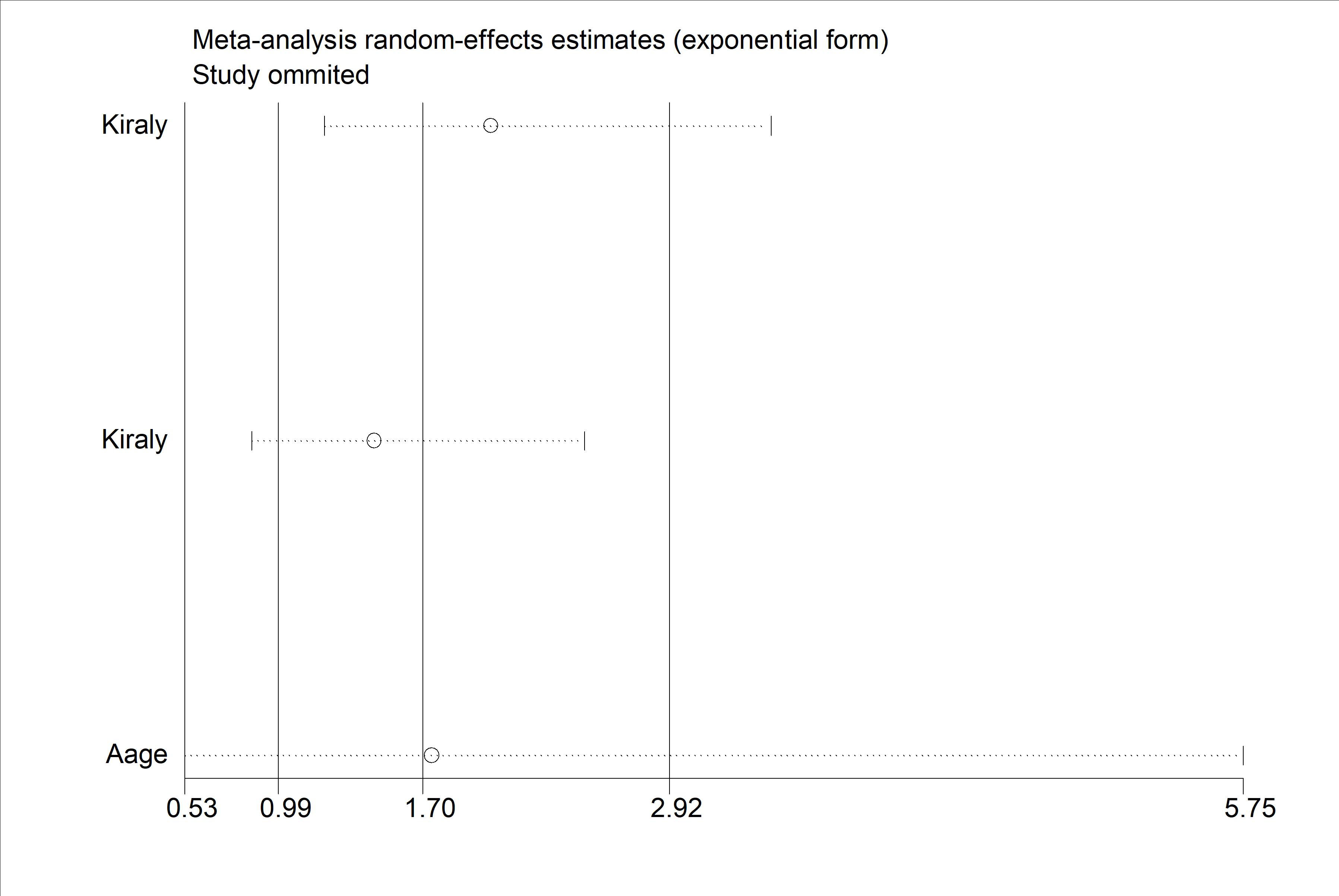

Supplement: Supplementary file 3 [file Image_2.jpg]

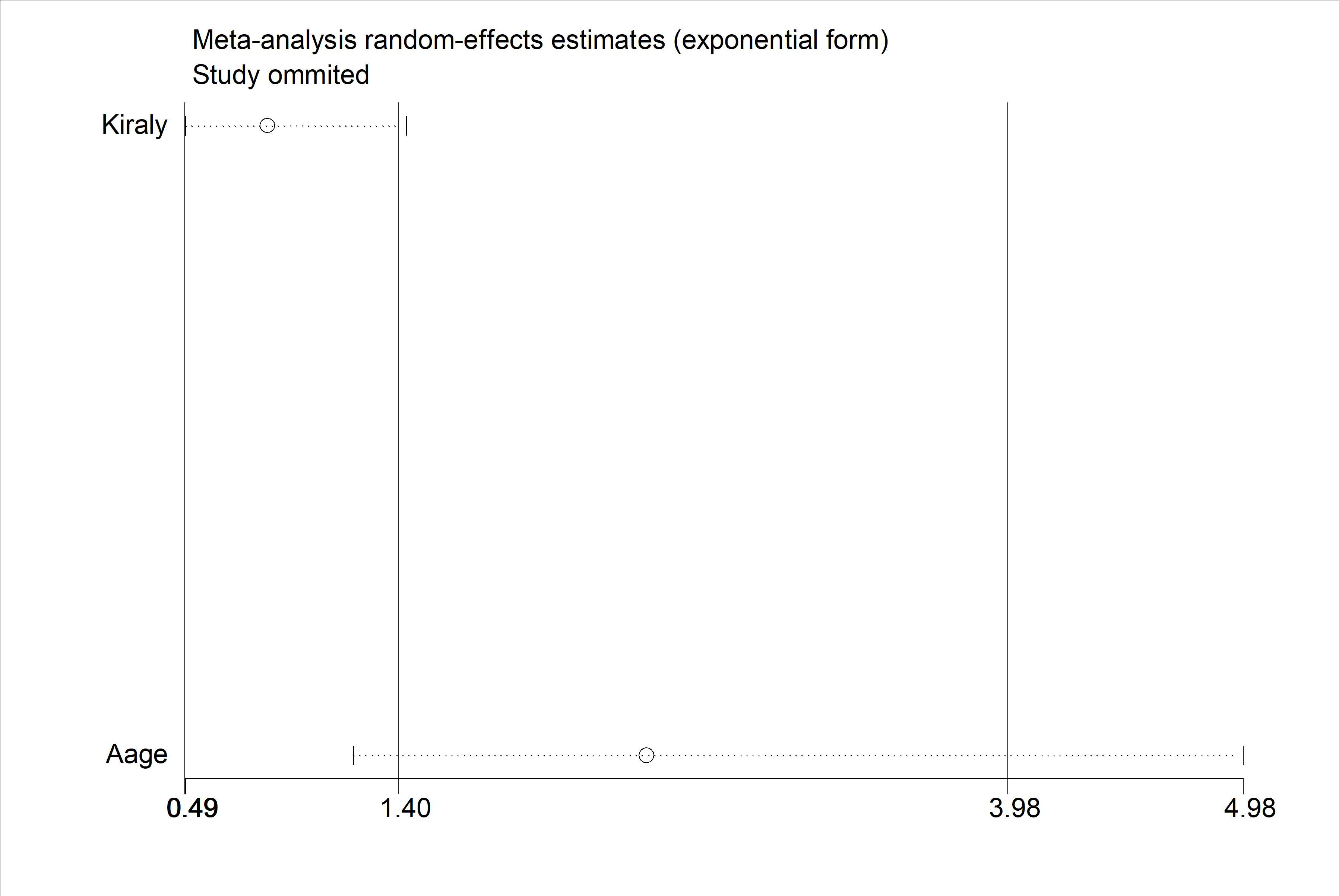

Supplement: Supplementary file 4 [file Image_3.jpg]

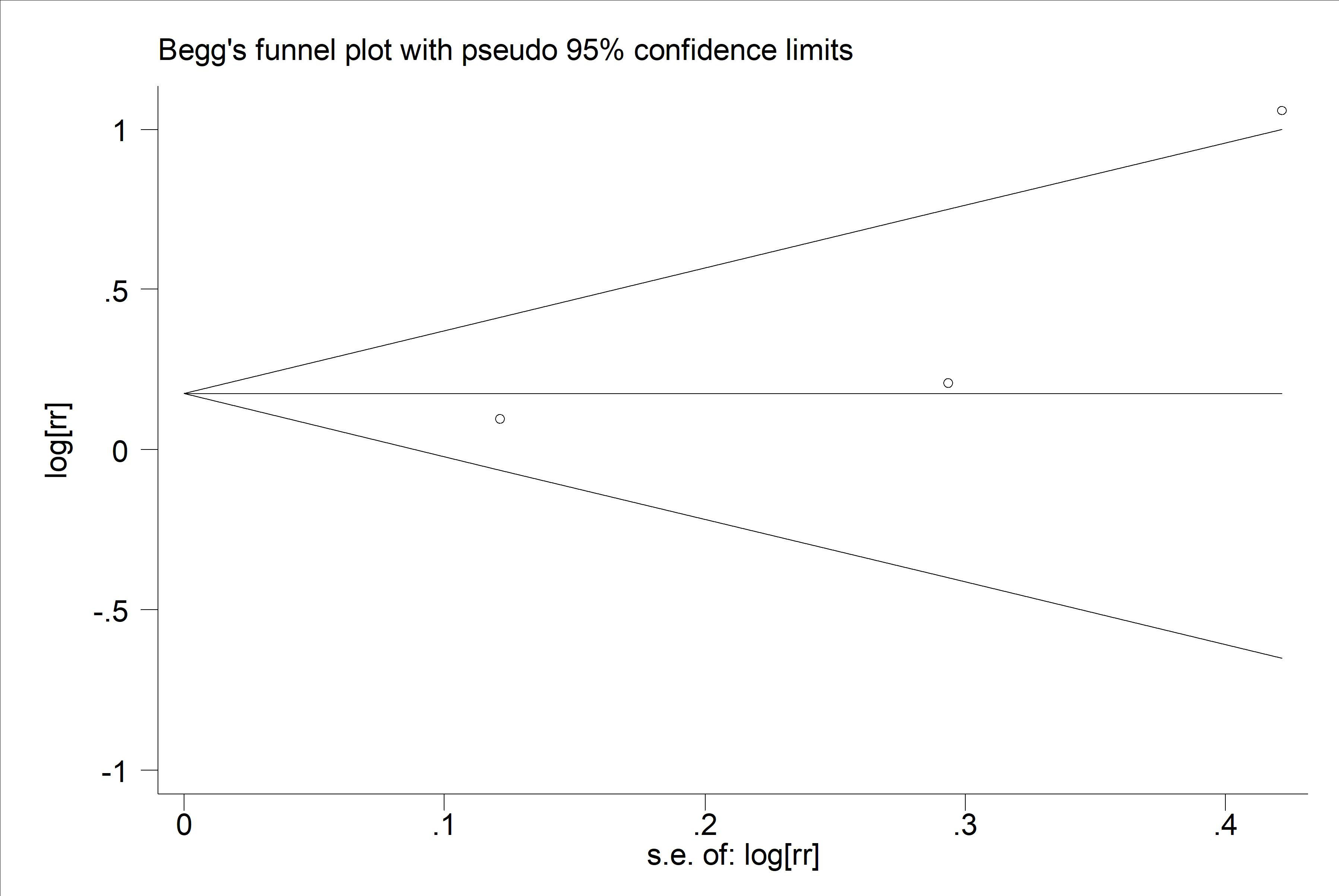

Supplement: Supplementary file 5 [file Image_4.jpg]

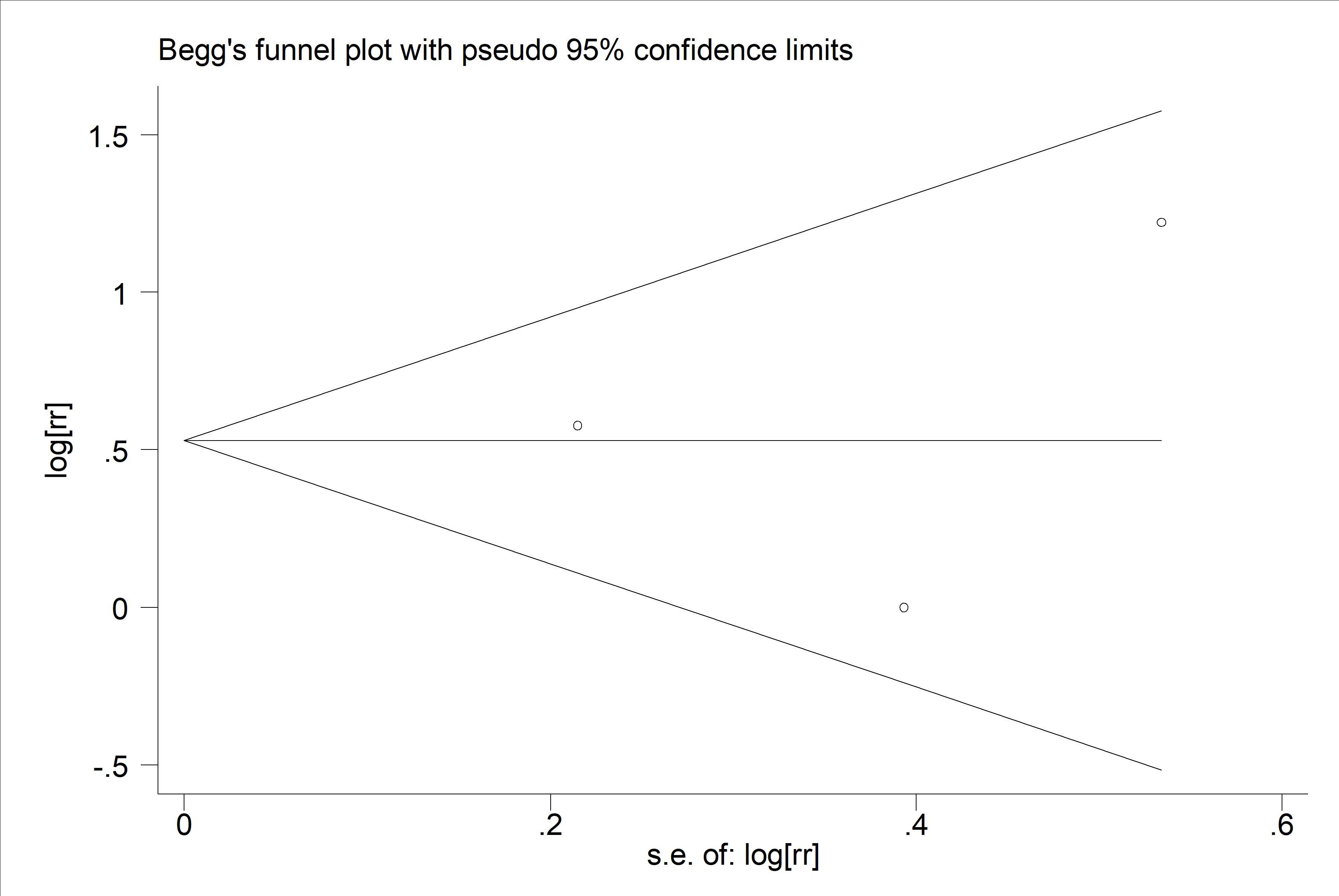

Supplement: Supplementary file 6 [file Image_5.jpg]

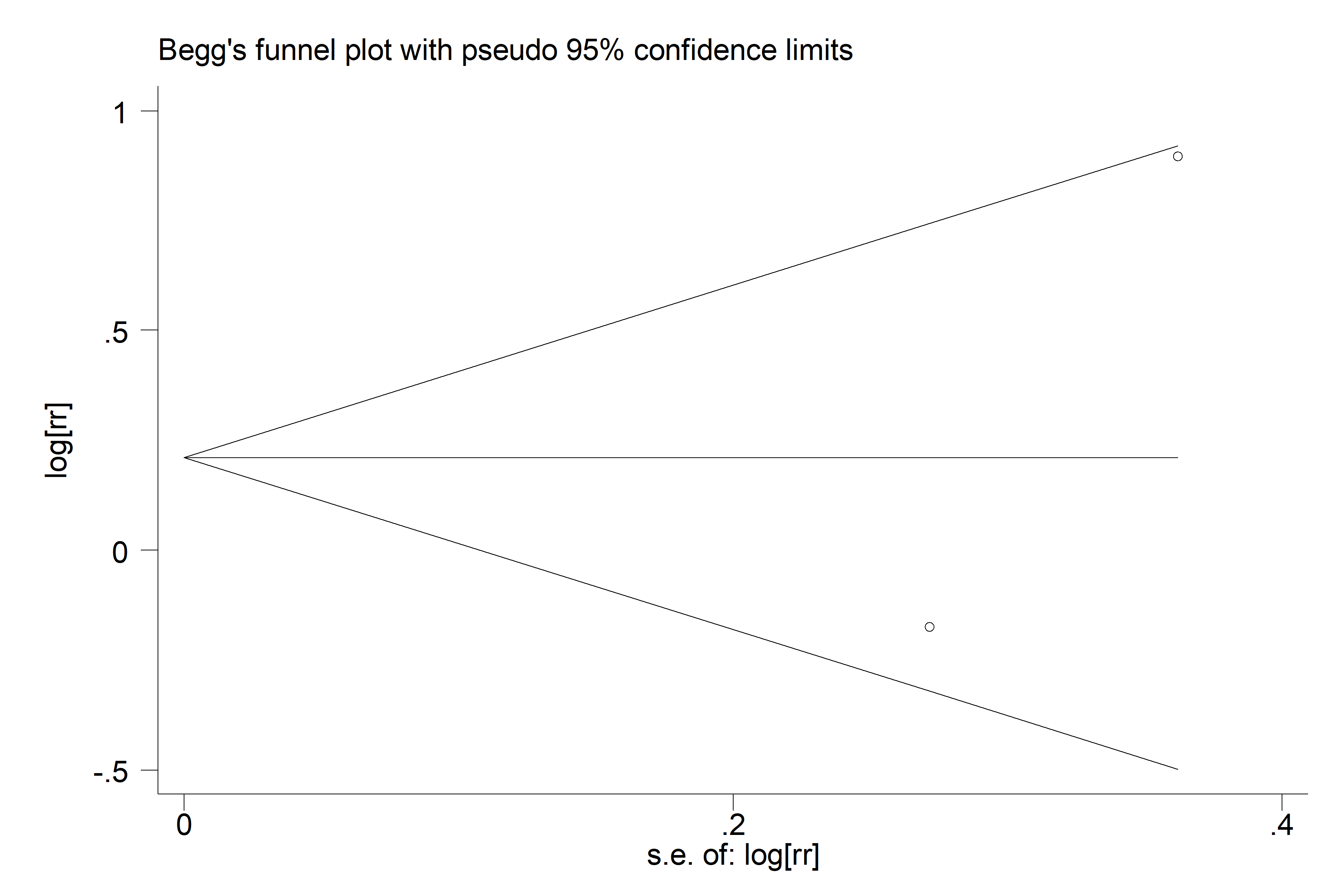

Supplement: Supplementary file 7 [file Image_6.png]
